# Population demography of feral honeybee colonies in central European forests

**DOI:** 10.1098/rsos.220565

**Published:** 2022-08-03

**Authors:** Patrick L. Kohl, Benjamin Rutschmann, Ingolf Steffan-Dewenter

**Affiliations:** Department of Animal Ecology and Tropical Biology, Biocenter, University of Würzburg, Würzburg, Germany

**Keywords:** pollinator decline, wild honeybees, beech forests, swarming, life-history traits, nest site selection

## Abstract

European honeybee populations are considered to consist only of managed colonies, but recent censuses have revealed that wild/feral colonies still occur in various countries. To gauge the ecological and evolutionary relevance of wild-living honeybees, information is needed on their population demography. We monitored feral honeybee colonies in German forests for up to 4 years through regular inspections of woodpecker cavity trees and microsatellite genotyping. Each summer, about 10% of the trees were occupied, corresponding to average densities of 0.23 feral colonies km^−2^ (an estimated 5% of the regional honeybee populations). Populations decreased moderately until autumn but dropped massively during winter, so that their densities were only about 0.02 colonies km^−2^ in early spring. During the reproductive (swarming) season, in May and June, populations recovered, with new swarms preferring nest sites that had been occupied in the previous year. The annual survival rate and the estimated lifespan of feral colonies (*n* = 112) were 10.6% and 0.6 years, respectively. We conclude that managed forests in Germany do not harbour self-sustaining feral honeybee populations, but they are recolonized every year by swarms escaping from apiaries.

## 1. Introduction

Honeybees are among the most widely known insects owing to their timeless cultural and economic value as a source of honey and wax [1]. As generalist flower visitors, they are pollinators of many wild and cultivated plants and managing their colonies is crucial for industrial crop production [2–4]. Today, *Apis mellifera* L. is usually seen as a domesticated species which needs to be maintained by humans to provide these services [5]. What is largely neglected in both science and practice, however, is that an unknown fraction of its global population is still made up of wild or feral colonies [6,7]. Wild-living honeybee colonies can complement managed ones both in providing ecosystem services (e.g. pollination; [8,9]) and disservices (e.g. competition with other species for food and nest sites; [10,11]), so they should be considered in population censuses [12]. Furthermore, wild-living honeybee populations can be a reservoir of native and/or locally adapted genes and therefore deserve conservation [13–18]. Finally, studying the life of honeybees in the wild can help understand basic aspects of the species' ecology, which in turn can be relevant for apiculture (reviewed by [19]).

The western honeybee is native to Africa, western Asia and Europe, and has been introduced to most other parts of the world [15,20]. In Africa, wild colonies are known to outnumber managed ones [21], and after their introduction in 1956, feral African honeybees also rapidly spread throughout (sub)tropical America [22,23]. In Europe and western Asia, in contrast, wild honeybee populations are considered extinct owing to the invasion of the ectoparasitic mite *Varroa destructor* [24] and its associated viruses [25,26]. Since no longitudinal population studies have been conducted in Europe itself, this assumption is based on studies of feral European honeybees within the species’ introduced range in North America which demonstrated initial drops in population sizes following the introduction of *V. destructor* [27–29]*.* However, it is not clear whether the parasite inevitably causes naïve wild-living honeybees to go entirely extinct because, on the population level, frequent reproduction by established colonies might level out colony losses [28–30]. There is a growing number of reports from Europe documenting the occurrence of honeybee colonies nesting wild in various types of cavities and habitats [7,14,17,31–36], but we currently lack detailed studies of their population dynamics.

To gauge the relevance of these wild-living honeybees, two basic questions need to be answered. On the one hand, we need robust information on their colony densities, how they vary between seasons and years, and how they relate to the densities of managed colonies. This is necessary to estimate how frequently they interact with other organisms and thus how much they matter for ecosystems [37]. On the other hand, we need to know whether wild-living honeybee colonies form self-sustaining populations or whether they are instead regularly founded by swarms that emigrate from apiaries [13]. Self-sustaining feral populations would be interesting subjects for the study of how honeybees manage to persist despite pressure by parasites [38,39]. Furthermore, knowing their population demography is necessary for rating the relative importance of apiculture versus near-natural habitat (e.g. woodland) in maintaining honeybee populations and their pollination services [35,36].

Answering the question of whether a given population is self-sustaining requires information on the annual survival and natality rates of its members [40,41]. Temperate-adapted honeybee colonies are sedentary and perennial cavity-nesters. They reproduce via colony fission in spring when the old queen and a number of young queens leave with swarms (daughter colonies) and the old nest is taken over by another young queen. Most wild-living colonies will reproduce annually starting after their first successful hibernation, with the average natality rate being around two swarms per colony per year [19]. Bees from managed colonies can also swarm and enter the feral population. Hence, a wild-living population can only be considered self-sustaining if the annual colony survival rate is high enough that losses can be compensated for by new swarms produced by the surviving wild-living colonies.

To determine the annual survival rate, one needs to make repeated surveys of their nest sites [30,42,43]. Unfortunately, finding an adequate number of colonies is often a cumbersome task. While ‘bee-lining’, the tracing of honeybees from artificial feeding sites to their homes, can be used as an unbiased search method [44], finding actual nests (and not only their approximate locations) is very time-consuming [7,32,44,45]. Hence, the most used method is asking the public for help [17,26,31,34,46,47]. The downside of citizen science is that the reported colonies are typically scattered over a large area, so that researchers further rely on many volunteers to collect data on survival rates, potentially compromising data quality. Moreover, honeybee nest sites detected via human search will naturally lead to a bias towards urban areas, where the densities of beekeeper-managed colonies are usually high [34]. However, feral honeybee colonies are ecologically more interesting in natural areas remote from human settlements. Often, these involve forests. Here we present a demographic study of feral honeybee colonies in German managed forests based on repeated surveys of cavity trees over a period of up to 4 years. We capitalized upon current maps of woodpecker cavity trees which are under protection from logging. Monitoring these trees allowed us to infer the regional densities and the temporal population dynamics of feral colonies and to determine their annual survival rates. These data, in turn, revealed whether the population was either self-sustaining or immigration-dependent.

## 2. Material and methods

### 2.1. Study areas and cavity trees

In forests managed for timber production, trees are rarely allowed to grow old enough to develop large holes through damage or decay [48,49]. Therefore, species that require large tree holes for nesting usually rely on cavities excavated by woodpeckers [48,50–52]. In German forests, black woodpecker (*Dryocopus martius* L.) cavities probably represent the main nesting opportunity for wild-living honeybees given that our inspection of woodpecker trees and a different, unbiased search method (bee-lining technique) yielded similar estimates of feral colony densities for (albeit different) forests [7]. We therefore assumed that surveying black woodpecker cavities would yield representative samples of the wild-living honeybee populations in managed forests.

**Figure 1.**
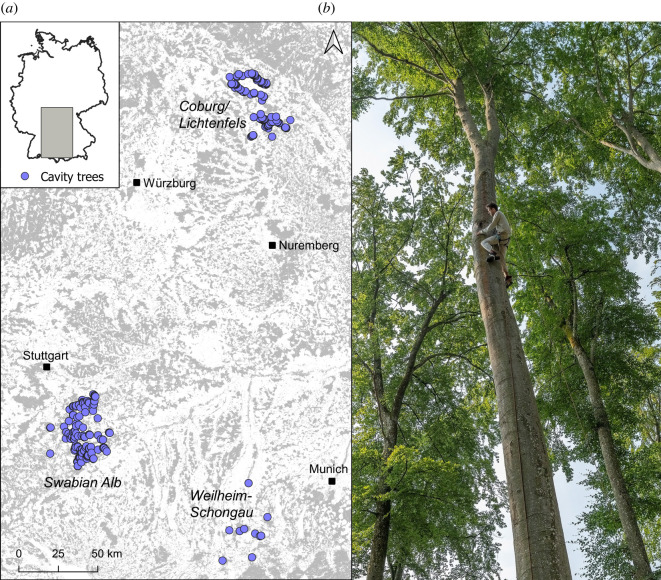
(*a*) Map of the cavity trees (blue dots) surveyed in three study regions in southern Germany. Forest areas are highlighted in grey (data from [53]) and the locations of four cities are indicated by black squares as reference points. (*b*) Photo of a typical black woodpecker cavity tree in the Swabian Alb, with one of the authors (B.R.) inspecting the nest entrance of a feral honeybee colony (note that cavities were inspected from the ground during standard inspections). Photo by Ingo Arndt.

We conducted our feral honeybee censuses in forests of three regions in southern Germany for which detailed maps of cavity trees (and the corresponding unique geographical coordinates) were available: in the Swabian Alb (Baden-Württemberg; [51]), in the counties of Coburg and Lichtenfels (north Bavaria; N. Wimmer 2019, personal communication) and in and around the county of Weilheim-Schongau (Bavarian Alpine Foreland; K. Zeimentz 2018, personal communication) (figure 1*a*). The forests were either dominated by beech (*Fagus sylvatica* L., the species that would naturally dominate most forests in Germany) or by Norway spruce (*Picea abies* L.*,* which is planted for timber production). The typical black woodpecker cavity tree was a large beech tree (greater than 98% of the considered cavity trees were beeches) with a diameter at breast height of 55 cm or more (figure 1*b*). The cavities were usually 10–12 m (range: 5–18 m) above ground level and had an entrance with a diameter of around 10 cm (range 5–15 cm) [54]. We had no information on the specific volumes of the cavities used in our survey but they probably held at least around 10 l, which is the approximate volume of freshly excavated black woodpecker cavities in beech trees [55].

In the Swabian Alb and in Coburg/Lichtenfels, we considered lists of 197 and 250 trees, respectively, which we inspected at least once during our study (see the electronic supplementary material for details on the selection of trees). The monitoring of many cavity trees in these two study regions allowed us to quantify cavity tree occupation rates and to estimate feral colony densities. In the third study region (Weilheim-Schongau), monitoring a large random sample of trees was not possible owing to time constraints. There, we specifically surveyed 14 cavity trees which were known to have been used by honeybees (K. Zeimentz 2018, personal communication). The observations from the third region were included in calculating feral colony survival rates. By collecting tree occupation and colony survival data from three distinct regions, we expected to obtain information that would adequately represent the status of feral honeybee populations in comparable settings (managed forests) across Germany and beyond.

### 2.2. Population monitoring

We inspected cavity trees three times per year in accordance with the honeybees' annual cycle of colony foundation, overwintering and reproduction. In July, after the main swarming season (in Germany: May and June [56]; B. Rutschmann, P. L. Kohl 2019, personal observations), we checked a high number of cavity trees to record the annual peak occupation rates. Between mid- and late September, we re-inspected those cavity trees which had been previously occupied to determine the late summer survival, which might be critical owing to the scarcity of floral resources [57]. Since new occupations were not to be expected between July and September, other trees were only inspected if they had not been checked before. From early to mid-April (before the swarming season), we determined the winter survival of all known honeybee colonies. Again, other trees were only checked if they had not been visited before. At all censuses, cavities were inspected with binoculars from the ground. We scored a cavity as being occupied if we observed bees that entered it and carried pollen (indicative of brood rearing activity in the nest). However, since recognizing pollen loads was not always possible owing to the height of the cavities, we also accepted regular and directional in- and outward flight traffic as a positive indicator. If we only saw individual bees performing erratic zig-zag flights around the entrance, which is typical for scout and robber bees, we did not consider the cavity as inhabited by a living colony. In the Swabian Alb, the population monitoring started in September 2017 [7], and in Weilheim-Schongau and Coburg/Lichtenfels, the surveys started in April and July 2019, respectively. We conducted the last systematic census in April 2021. Colonies alive at that date were re-inspected once more in July 2021. For the Swabian Alb and for Coburg/Lichtenfels, we estimated feral population densities for each sampling date based on the cavity occupation rates and the known densities of the inspected trees (see the electronic supplementary material for details).

### 2.3. Genotyping bees to assess colony continuity

We could directly infer the summer and winter survival rates of feral honeybee colonies from the observed proportion of colonies alive in autumn and after winter, respectively. However, when a cavity was occupied both before and after the swarming season, this did not necessarily prove that the original colony had survived the spring. This is because nest sites which become vacant through colony death in spring can be quickly re-occupied by new swarms. To estimate the rate at which we would incorrectly note spring survival when the original colonies had actually died, we analysed the genetic relatedness of bees from a random subset of the cavities that had been occupied both before and after the swarming season.

For 4–18 workers and/or drones per colony, we determined the DNA fragment lengths of 12 microsatellites representing a subset of the markers proposed by Shaibi *et al*. [58] at the Institute of Human Genetics of the University of Würzburg using a capillary sequencer (see the electronic supplementary material for details). We inferred the genotypes of the colonies’ queens with the aid of the program ‘COLONY’ [59] and calculated coefficients of relatedness [60] between pairs of queens inhabiting the same tree at different time points using the package ‘related’ for R [61]. In case the relatedness between queens was at least 0.25 (grandmother–granddaughter relationship), we considered that the colony was the same before and after the swarming season, and thus that it had survived the spring.

### 2.4. Estimating demographic parameters

We obtained the colonies' annual survival rate (*s*) by multiplying the observed summer, winter and spring survival rates. Based on published data on the probability of reproduction and the average number of swarms produced in temperate-adapted European honeybee colonies, we assumed that the average natality rate (*n*) in our population would be two swarms per feral colony per year (range: 0–4; [30,62–64]; see the electronic supplementary material). Based on the annual survival and natality rates, we calculated the net reproductive rate (*R*_0_) [40,65], which describes how the population of feral colonies would change from year to year if no immigration of swarms from managed hives occurred:

$$R_{0}=s+s\times n,$$

where a value of *R*_0_ ≥ 1 indicates that the population is self-sustaining or expanding, while a value of *R*_0_ < 1 indicates that it is dependent upon immigration. Another clear indicator of the population status of the feral colonies is the number of swarms (daughter colonies) that each colony would need to produce annually for the population to be self-sustaining (*D*). This statistic is easily interpreted when compared to the assumed natality rate of two swarms per colony per year (modified after [43]):

$$D=\;\frac{1-s}{s}.$$

Both statistics are based on the following assumptions:

(i) the feral colonies we considered for determining the annual survival rate represented a random sample. This is reasonable, since there are very few other cavities available in German forests apart from the woodpecker cavities that we surveyed (see above);
(ii) most swarms produced by the feral colonies were able to find a new nest site. This was probably the case since most cavities were still vacant after the swarming season;
(iii) most of the newly founded colonies survived the first weeks between their swarming event in May or June and our summer survey in July, i.e. we kept track of most colonies that ever colonized the trees monitored during the study period;
(iv) colonies were unlikely to migrate from one tree to another without colony fission (in which case we would have noted the ‘death’ of a nest although the number of colonies in the population stayed constant). This assumption is legitimate because simple colony migration or absconding is very rare in temperate-adapted honeybees [66]; and
(v) swarms produced by feral colonies stayed part of the feral population. This assumption is valid, since neither do beekeepers usually install bait hives, nor are they likely to find and directly capture swarms issued by wild-living colonies in forest areas.

The average lifespan (*L*) of feral colonies can be estimated by summing over a range of age classes the products of each age (in years) and the probability of dying at that age:

$$L=\overset{10}{\underset{A=0}{\sum }}[A+0.5][s^{A}][1-s],$$

with *A* being the colony age in the number of completed years and *s*, as above, being the annual survival probability for all colonies. With the use of this formula, it is assumed that colonies die, on average, halfway through a year (e.g. colonies which died within their first year are assigned a lifespan of 0.5 years), and that the annual survival probability is constant regardless of colony age. The latter can be justified by the fact that colonies are regularly taken over by young queens (during swarming or natural queen supersedure), so that colony lifespan is not restricted by queen longevity. However, the nests of honeybees age over time since they do not renew their beeswax combs, and this has possibly negative effects on colony development [67,68]. As a solution to avoid overestimating lifespan, we arbitrarily restricted the above summations to a maximum age class of *A* = 10, expecting that few colonies will ever live more than 10 years. Demographic studies of feral honeybees in the northeastern USA [30,42] and in southeast Australia [43] found that newly founded colonies have a significantly lower survival probability than established colonies aged at least 1 year. In such a case, the formula needs to be adapted (see the electronic supplementary material).

### 2.5. Statistical analyses

In calculating summer, winter and spring survival rates, we pooled all colony observations across years and study regions. Since observations were not equally distributed over time and space, this procedure would have led to a bias in the estimates if survival rates had differed strongly between years or regions. However, this was not the case, so we believe that the reported survival rates are accurate. We used two-sided Fisher's exact tests (in the case of two samples) and *χ*^2^-tests (in the case of more than two samples) to test for differences in the proportions of surviving colonies or for differences in the proportions of occupied trees. All statistical tests were performed in R v. 4.0.5 [69]. Figures were created using QGIS v. 3.16.8 [70] and with the R-package ‘ggplot2‘ [71].

## 3. Results

### 3.1. Cavity tree occupation and population dynamics of feral honeybee colonies

**Figure 2.**
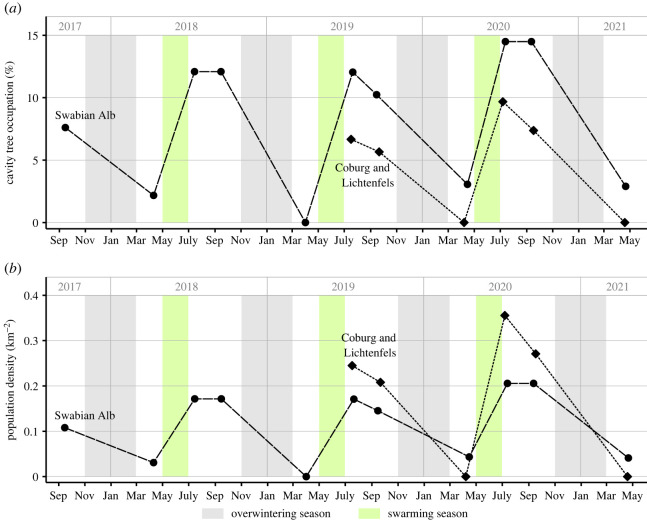
Temporal population fluctuations of feral honeybee colonies in forests of the Swabian Alb (September 2017–April 2021) and in the counties Coburg and Lichtenfels (July 2019–April 2021). The first data point (Swabian Alb, September 2017) has previously been reported [7]. (*a*) Percentage of cavity trees occupied by feral honeybee colonies. See the electronic supplementary material, table S2 for an overview of the numbers of colonies and trees considered. (*b*) Minimum population density of feral honeybee colonies as inferred from cavity occupation rates and the known densities of cavity trees.

The monitoring of cavity trees in the Swabian Alb and in Coburg/Lichtenfels revealed a recurring temporal pattern of population fluctuations: feral colony numbers peaked in summer, decreased moderately until autumn, dropped massively during winter and recovered during the swarming season in spring (figure 2). In July, mean cavity occupation rates were 12.9% in the Swabian Alb (range: 12–14.5%, three summers 2018–2020) and 8.2% in Coburg/Lichtenfels (6.7% in 2019 and 9.7% in 2020). In September, average occupation rates were 11.1% in the Swabian Alb (four autumns, 2017–2020) and 6.5% in Coburg/Lichtenfels (two autumns, 2019 and 2020). In April, occupation rates had dropped to average values of 2% in the Swabian Alb (range 0–3.1%, four springs, 2018–2021) and 0% in Coburg/Lichtenfels (two springs, 2020 and 2021). Translating occupation rates into population densities revealed mean values of 0.18, 0.16 and 0.03 colonies km^−2^ for the Swabian Alb and 0.30, 0.24 and 0 colonies km^−2^ for Coburg and Lichtenfels for July, September and April, respectively (figure 2*b*). When averaging across years and study regions, the expected summer occupation rate and the respective maximum population density were 11% and 0.23 colonies km^−2^, respectively. Accordingly, the minimum occupation rate and population density (after winter) were around 1.4% and 0.02 colonies km^−2^.

Not only did the seasonal changes in tree occupation rates follow a predictable pattern, the spatial distribution of feral colonies did too: trees that had been occupied in the previous year but had become vacant during the winter were five to 15 times more likely to be recolonized by new swarms than trees without recent bee occupation. This was revealed when considering cavity (re-)occupations in years in which all cavities had become free of bees during winter. Regarding the Swabian Alb, of the 11 trees which housed bees in summer 2018, 63.6% (seven trees) were re-colonized in 2019, while of the 79 trees that were not occupied in 2018 but reinspected in 2019, only 13.9% (11 trees) were colonized. In Coburg and Lichtenfels, 64.2% (nine out of 14) of the trees that were occupied in 2019 were re-colonized in 2020, while only 4.1% (seven out of 169) of the trees that were not occupied in 2019 were colonized in 2020. These differences in occupation probabilities of former bee trees and non-bee trees were highly significant (*p* < 0.001 for both Swabian Alb and Coburg/Lichtenfels, Fisher's exact tests).

### 3.2. Population demography of feral honeybee colonies

We gathered data on feral colony survival from a total of *n* = 112 individual colonies from three woodland regions. While 90% of feral colonies survived the summer (July–September; *n* = 100 observations), only 16% survived the winter (September–April; *n* = 81). Considering spring survival (April–July), we found a total of 23 trees to be colonized in early spring, of which 19 (82.6%) were still occupied in summer (‘apparent’ spring survival). In nine of these 19 cases, we were able to genotype bees sampled from the colonies before and after the swarming season to determine the relatedness of their mother queens. In eight out of the nine cases (88.9%), the queens were closely related (at least mother–daughter relationships; see the electronic supplementary material, tables S7.1–8), indicating that the respective trees had been continuously colonized by the same colonies. We therefore estimate that the actual spring survival rate was 0.826 × 0.889 = 0.724 or 72.4%. The summer, winter and spring survival rates did not differ significantly between regions, nor between years (*p* > 0.05, *χ*^2^-tests; see the electronic supplementary material, tables S3–S5), nor between founder colonies (aged less than 1 year) and established colonies (aged at least 1 year) (*p* > 0.3, Fisher's exact tests; see the electronic supplementary material, table S6).

The annual survival rate of feral colonies resulting from the product of summer, winter and spring survival rates is *s* = 0.106 or 10.6%. Consequently, each colony would need to produce an average of *D* = 8.43 swarms annually to maintain the population. This clearly exceeds the assumed natality rate of *n* = 2 swarms produced per colony per year. Accordingly, the net reproductive rate of the feral honeybee population is *R*_0_ = 0.318, indicating that it is currently not self-sustaining. The estimated average lifespan of feral colonies in German forests is 0.619 years.

## 4. Discussion

Despite the potential relevance of wild-living honeybee colonies in complementing managed colonies, until now, detailed studies on their population dynamics have been lacking in Europe. We conducted a demographic study to clarify the population status of feral honeybees in Germany. Our results show that feral honeybee colonies populate forests at densities of about one colony in 4–5 km^2^ each summer, but that they do not form self-sustaining populations.

This conclusion is grounded on the result that only about one out of 10 feral colonies survived annually, meaning that successful colonies would need to produce eight–nine daughter colonies each swarming season for the population to be stable on its own. However, since temperate-adapted honeybee colonies only produce two swarms on average per year, we infer that the feral population would decrease if there was no immigration of foreign swarms. That immigration is indeed occurring every spring is evident, because the summer population densities of feral colonies varied little throughout the years of our study. In quantitative terms, we estimate that each year, around 70% of the forest-dwelling feral population must be recent immigrants (derived from the complement of its net reproductive rate, 1 − *R*_0_). The most likely source of these immigrants is the population of colonies managed by beekeepers in apiaries.

In recent years, several studies have reported on the occurrence of wild-living honeybee colonies in Europe [7,14,17,31,32,34–36]. In the few cases where colony densities were estimated, the numbers were comparable to those reported here (rural avenues in Poland [14]: 0.1 km^−2^; Hainich National Park, Germany [7]: 0.13 km^−2^; agricultural landscape in northwest Spain [36]: 0.17–0.22 km^−2^). In turn, all known feral populations which are evidently self-sustaining exhibit significantly higher colony densities: at least around 1 colony km^−2^ in temperate regions [19], and often greater than 5 colonies km^−2^ in (sub)tropical regions [12,72,73]. Therefore, it seems likely that in many of the European cases wild-living colonies might merely represent recent escapees from apiaries. Importantly, our observations confirm the known habit of honeybee swarms to prefer cavities that have been used by bees before [74], meaning that reports about cavities that house wild honeybees for multiple years do not necessarily demonstrate that individual colonies live that long [17,34]. For example, a recent study from Ireland [17] suggests that feral colonies commonly survive for 2–3 years, which would be indicative of a viable feral population (table 1). Unfortunately, it is unclear whether the reported survival times refer to colony lifespans or to the number of consecutive years a nest site was inhabited. Without robust estimates of colony survival rates, the status of a given population of feral colonies remains ambiguous.

**Table 1.** Demographic parameters of three populations of feral honeybee colonies. (Information is provided on the location of the populations, the annual survival rates of feral colonies (either for all colonies, *s*, or for founder and established colonies separately, *f* and *e*), the average lifespan of feral colonies (*L,* in years), the number of swarms needed to be produced per colony and year for the population to be self-sustaining (*D*), and the net reproductive rate of the populations (*R*_0_).)

	*s*					
population	(*f*)	[*e*]	*L*^a^	*D*^b^	*R_*0*_*^b^	reference
Arnot Forest, USA	(0.24)	[0.79]	1.34	0.94	1.55	[30,42]^c^
Wyperfield National Park, Australia	(0.32)	[0.76]	1.53	0.85	1.62	[43]
German forests	0.11		0.62	8.43	0.32	this study

^a^The average colony lifespan of the Arnot forest and Wyperfield populations deviate from what was reported in the original studies since we used a modified calculation (see the electronic supplementary material).

^b^To calculate *D* and *R_0_* in the case of the Arnot forest and the Wyperfield populations, we considered as the annual survivorship of all colonies (*s*) the mean of the survival rates of founders (*f*) and established colonies [*e*].

^c^Seeley [30] presents in the Appendix 1 of his paper an overview of the number of feral colonies that survived and died during his population studies in the 1970s and in the 2010s. He distinguished between summer and winter survival and between founder and established colonies. We used these data to calculate average annual survival rates for founder and established colonies.

Two other populations of feral European honeybees, from the Arnot forest in the northeastern USA [19,30,42] and from the Wyperfield National Park in southeast Australia [43], have been investigated with respect to their demography (table 1). The colony survival rates in these populations are around five times higher than in the German population (average survivorship greater than 50% versus 11%), which is enough for them to be self-sustaining or even expanding. Furthermore, in the USA and Australia, colonies older than 1 year (established colonies) have a significantly higher annual survival probability than colonies younger than 1 year (founder colonies). This can be explained by the extra amount of energy that is needed for the foundation of a new nest, which involves building beeswax comb and food reserves from scratch [42]. In German forests, in turn, feral colonies had low survival rates regardless of their age. This suggests that either food availability is so low that not even established colonies can acquire enough, or that other factors are limiting colony survival. Regarding forage availability, the forests we worked at probably do not offer many nectar and pollen sources, since they are dominated by single wind-pollinated tree species (beech or spruce). By contrast, the deciduous forests in northeastern USA usually contain several insect-pollinated tree species (e.g. *Acer* spp., *Tilia americana*) [19] and the *Eucalyptus* woodlands in southeast Australia produce abundant nectar and pollen [43]. Furthermore, tree cavity densities are higher in Australian and North American forests compared to European forests [48], so that forest-dwelling feral honeybees probably have more (and more diverse) nesting opportunities. Although we found that about 90% of the existing black woodpecker cavities were still vacant each summer, suggesting that the cavity density *per se* is not limiting, perhaps the cavities themselves are not optimal for honeybees. For example, they might be too small to hold sufficient food stores for the winter, or too difficult to defend and thermoregulate given the relatively large entrance holes [75]. The fact that feral swarms preferentially occupied certain ‘bee trees’ indeed suggests that many of the inspected black woodpecker cavities were not even attractive to the bees in the first place. Besides these ecological factors, the three investigated populations differ by an evolutionary factor. Both in the Arnot Forest and at Wyperfield National Park, feral colonies outnumber managed ones [43,76]. Therefore, their populations can adapt evolutionarily to a life in the wild. In Germany, in contrast, the density of feral colonies is much lower than the density of managed ones (see below), so that the regional honeybee population is mainly shaped by the selection pressures prevailing under beekeeping management [18]. Today's most obvious selection pressure for wild-living honeybee populations, which is attenuated in apiculture, is the infestation by the parasitic mite *V. destructor* [77]. Indeed, there is evidence that feral honeybee populations from the northeastern USA differ genetically from sympatric managed populations, and these differences are likely to involve adaptations which balance their relationship with *V. destructor* and its associated pathogens [76,78,79]*.* However*,* the feral colonies living in German forests have only recently left the apiary, meaning that they are unlikely to be genetically distinguishable from managed colonies and equally unequipped against the parasite.

Although feral honeybees in German forests are unlikely to bear genetic adaptations to parasites, they might still be relevant with respect to their effects on ecosystems. We found average population densities of 0.23 colonies km^−2^ from early summer onwards. This number exceeds our previous estimate of 0.11 feral colonies km^−2^ for managed beech forests in the Swabian Alb [7] because the latter was based on a census made in September 2017, when a fraction of that year's population had probably already died, and because the feral population density was generally lower in the Swabian Alb (0.18 colonies km^−2^ in summer) than in our second reference region, Coburg/Lichtenfels (0.30 colonies km^−2^). Under the assumption that our estimate of one colony in 4–5 km^2^ approximately represents the feral colony density across the wider countryside in southern Germany, and considering that the average density of managed honeybees is around 4 colonies km^−2^ (Baden-Württemberg: 5.21 km^−2^, Bavaria: 2.85 km^−2^ [80]), then feral colonies make up about 5% of the total honeybee population on a country-wide scale. However, after winter, when the feral populations have dropped to densities of only about one colony in 50 km^2^, their share is much smaller (around 0.5%). At the local scale, the population density of feral honeybees will depend on the availability of cavities and the number of managed colonies within the dispersal range of swarms. For example, feral colonies should be relatively rare in intensive agricultural areas owing to the scarcity of nest sites, unless rural avenues are lined with hollow trees [14]. Therefore, we can expect that swarms issued by managed hives in farmland typically disperse into nearby forests, if available. Indeed, the feral colonies we surveyed in our study must have almost exclusively stemmed from managed colonies in adjacent crops, grasslands, orchards or villages. In cities, in turn, swarms escaping from managed hives are likely to find many nesting opportunities, whether it be cavities in old-grown trees in parks or hollow spaces in man-made structures [17,34], so that managed and feral colonies will live spatially intertwined.

Our study showcases that the feralization [81] of honeybees is much more common than previously assumed. Germany-wide, tens of thousands of swarms will emigrate from apiaries each spring to found feral colonies in tree holes or other cavities. Therefore, it is imprecise to consider the honeybee population as fully managed or domesticated, and it needs to be recognized that the impact of beekeeping on the environment goes beyond the effect of bees foraging in the area around apiaries. Whether beekeepers' incidental ‘service’ of issuing feral swarms to the surroundings is generally beneficial or not is currently unclear [37]. Questions remain about whether low versus zero abundances of feral honeybees affect the pollination of wild plants in forests [2], how honeybees interact with other organisms in tree cavities [10,51,82] and whether feral honeybees play a role in the transmission of parasites and pathogens to managed honeybees and non-*Apis* bees [26,47,83–86]. Furthermore, with the goal to improve the wellbeing of all honeybees, it is important to know why feral colonies currently fail to establish self-sustaining populations, whether it be owing to ecological (e.g. lack of floral food resources and suitable nesting sites, parasite pressure) or evolutionary factors (domestication).

## Data Availability

The data used in this paper is provided in the electronic supplementary material [87].
